# Nucleophilic Aromatic Substitution of Pentafluorophenyl-Substituted Quinoline with a Functional Perylene: A Route to the Modification of Semiconducting Polymers

**DOI:** 10.3390/polym15122721

**Published:** 2023-06-18

**Authors:** Stefania Aivali, Konstantinos C. Andrikopoulos, Aikaterini K. Andreopoulou

**Affiliations:** 1 Department of Chemistry, University of Patras, University Campus, GR26504 Rio-Patras, Greece; k.andrikopoulos@upnet.gr; 2 Département de Chimie, Université Laval, Quebec City, QC G1V 0A6, Canada

**Keywords:** perylene diimide, nucleophilic aromatic substitution, post-polymerization modification, perflurophenyl semiconducting poly(quinolines), optoelectronic properties

## Abstract

A systematic study of the influence of the chemical substitution pattern of semiconducting polymers carrying side chain perylene diimide (PDI) groups is presented. Semiconducting polymers based on perflurophenyl quinoline (5FQ) were modified via a readily accessible nucleophilic substitution reaction. The perfluorophenyl group was studied as an electron-withdrawing reactive functionality on semiconducting polymers that can undergo fast nucleophilic aromatic substitution. A PDI molecule, functionalized with one phenol group on the bay area, was used for the substitution of the fluorine atom at the para position in 6-vinylphenyl-(2-perfluorophenyl)-4-phenyl quinoline. The final product was polymerized under free radical polymerization providing polymers of 5FQ incorporated with PDI side groups. Alternatively, the post-polymerization modification of the fluorine atoms at the para position of the 5FQ homopolymer with the PhOH-di-EH-PDI was also successfully tested. In this case, the PDI units were partially introduced to the perflurophenyl quinoline moieties of the homopolymer. The para-fluoro aromatic nucleophilic substitution reaction was confirmed and estimated via ^1^H and ^19^F NMR spectroscopies. The two different polymer architectures, namely, fully or partially modified with PDI units, were studied in terms of their optical and electrochemical properties, while their morphology was evaluated using TEM analysis, revealing polymers of tailor-made optoelectronic and morphological properties. This work provides a novel molecule-designing method for semiconducting materials of controlled properties.

## 1. Introduction

Perylene diimide (PDI) is among the most studied organic molecules for organic opto-electronic applications [1,2]. Thanks to its outstanding properties, such as its strong n-type character proving low LUMO levels, its powerful absorption at 400 and 650 nm, which can be tunable, and its good thermal and chemical stability, PDI has been used a myriad of times for the construction of new small molecules or polymeric structures, achieving exceptional results [3,4,5]. From a synthetic point of view, many efforts were made to tailor PDI’s characteristics by utilizing the various positions that its structure provides for modification (imide groups, ortho and bay areas of the perylene skeleton) [6,7]. Complicated three-dimensional structures combining different organic building blocks were investigated in order to synthesize non-planar, PDI-based molecules, acting as non-fullerene acceptors for organic photovoltaics (OPVs) [8,9,10,11,12,13,14,15]. Generally, in these systems, well-known π-conjugated donor–acceptor type copolymers are developed through typical palladium-catalyzed synthetic procedures such as Stille, Suzuki, and Heck-type polymerizations [16,17], and PDI is usually incorporated as one of the acceptor building blocks of the polymeric structure [18,19,20,21,22,23,24]. Notwithstanding their usability and wide substrate scope, these reactions may require tedious synthetic methodologies and purification processes. Hence, alternative approaches were reported for organic semiconductors minimizing the toxic byproducts [25,26]. Regarding the PDI-based polymers, little progress has been made for polymers using PDI as side chain, with limited examples in the field [5,27,28,29]. These examples mainly involve styrenic [30] or acrylic [31,32] backbones, where PDI derivatives are usually grafted as side chains. In an effort to isolate the PDI moiety and transfer its optical properties to the solid thin film state, in the majority of these examples, the PDI moiety is isolated via long alkyl chains. Another example of side-grafted polymers involves the use of a polynorbornene backbone, with applications in OFETs [33] and OLEDs [34].

Metal-free [3+2] cycloaddition reactions, Diels–Alder reactions, and thiol–alkene radical addition reactions are rising as greener click reactions owing to their high yield and simple synthetic procedures [26,35,36,37]. The perfluorophenyl group (PFP) is of great interest as a low-cost functionality for the development of complex functional materials [38,39,40]. It can easily react with organic materials or surfaces due to the strong electron-deficient nature provided by the five fluorine bonds [35,41]. More specifically, it undergoes nucleophilic aromatic substitutions with a variety of nucleophiles (alcohols, amines, phosphates, and thiols) offering the possibility of the controlled modification of fluorine atoms, and providing favorable solubility [42,43]. The perflurophenyl group has also been studied in semiconducting polymers such as poly(3-hexyl thiophene) P3HT for controlled functional semiconducting materials [44,45,46,47,48]. These reasons highlight the PFP group as a promising moiety for the activation of various molecules and macromolecules.

Previously, we developed hydroxyphenyl-PDI molecules by attaching hydroxyphenyl groups to both or to one of the bay positions of the PDI core [47,48]. These hydroxyphenyl-PDIs were used for the substitution of perflurophenyl quinoline-based small molecules to afford ether-based, PDI-quinoline electron accepting small molecules [47]. In a second case, the di-hydroxyphenyl-PDI monomer was polymerized with aliphatic dibromides, producing rigid–flexible aromatic–aliphatic polyethers [48]. Moreover, some of our groups’ earlier efforts were devoted to electron-accepting perfluorophenyl-quinolines combined with single-wall carbon nanotubes (SWCNTs) or fullerene derivatives (C_60_, C_70_, PCBM), as electron-accepting hybrid additives for OPVs [49,50,51].

Herein, taking these past efforts further, a mono hydroxyphenyl-PDI molecule was employed as a functional entity for the modification of perfluorophenyl moieties to create side PDI-decorated polymers. For this reason, the hydroxyphenyl-PDI reacted under nucleophilic substitution with 6-vinylphenyl-(2-perfluorophenyl)-4-phenyl quinoline (vinyl-5FQ) [49]. This new vinyl monomer was polymerized through free radical polymerization (FRP), creating a poly(PDI-quinoline) homopolymer. In addition, PDI-decorated poly(quinoline)s were created via a post-polymerization modification route, using the mono hydroxyphenyl-PDI molecule as a nucleophilic reagent. The post-polymerization modification provided partially modified poly(quinoline)s appended with PDI units. The substitution reaction was affirmed via ^1^H and ^19^F NMR spectroscopies. The vinyl-Ph5FQ-PDI monomers and the final polymers created via the two different approaches were characterized, concerning their optical, electrochemical, and morphological properties. Notably, distinct differences in the behavior of the fully substituted P5FQ homopolymer versus the partially PDI-incorporated copolymers were revealed.

## 2. Materials and Methods

### 2.1. Materials

6-vinylphenyl-(2-perfluorophenyl)-4-phenyl-quinoline (vinyl-Ph5FQ) [49], homopolymer PPh5FQ [49], and hydroxy phenyl di-(2-ethylhexyl)-perylene diimide PhOH-diEH-PDI [47] were synthesized according to reported procedures. All other solvents and reagents were purchased from Merck and were used without further purification unless otherwise stated.

### 2.2. Synthetic Procedures

#### 2.2.1. Synthesis of Vinyl-Ph5FQ-PhO-di-EH-PDI

In a 25 mL flame-dried round-bottom flask filled with argon and equipped with a reflux condenser and a magnetic stirrer, PhOH-diEH-PDI (400 mg, 0.566 mmol), vinyl-Ph5FQ (375 mg, 0.792 mmol), and K_2_CO_3_ (178 mg, 2.376 mmol) were dissolved in 8 mL DMF_dry_. The flask was evacuated and filled with argon several times. The reaction mixture was vigorously stirred at 90 °C for 4 h under argon. The mixture was rotary evaporated to diminish the DMF solvent, and the resulting mixture was precipitated in a mixture of methanol and deionized water. The solid was filtered and washed with deionized water and methanol. The crude solid was further purified via column chromatography (CHCl_3_, silica gel). Yield: 0.565 g (85%). ^1^H NMR (600 MHz, CDCl_3_): δ (ppm) = 8.69–8.65 (d, 1H), 8.65–8.61 (d, 1H), 8.59–8.52 (m, 3H), 8.34–8.30 (d, 1H), 8.22–8.18 (m, 2H), 8.09–8.05 (dd, 1H), 7.90–7.86 (d, 1H), 7.65–7.61(m, 4H), 7.61–7.52 (m, 4H), 7.51–7.44 (m, 4H), 7.41–7.39 (m, 1H), 7.25–7.20 (d,1H), 6.80–6.70(q, 1H), 5.86–5.82 (d,1H), 5.35–5.25 (d,1H), 4.20–4.04(m, 4H), 2.09–2.01 (m, 2H), 1.90–1.71 (m, 16H), 1.03–0.95 (m, 12H); ^19^F NMR (564 MHz, CDCl_3_): δ (ppm) −153.48, −142.45. ^13^C NMR (150 MHz, CDCl_3_): δ (ppm) = 163.84, 163.81, 163.64, 163.57, 157.45, 149.6, 148.2, 146.9, 144.3, 142.6, 140.9, 140.6, 140.0, 139.6, 138.1, 137.4, 137.3, 136.2, 136.0, 134.9, 134.6, 134.4, 133.7, 132.6, 131.1, 130.9, 130.6, 130.2, 129.9, 129.6, 129.0, 128.9, 128.8, 128.3, 128.0, 127.6, 127.5, 126.8, 126.3, 123.5, 123.46, 123.3, 123.0, 122.7, 122.4, 122.3, 117.7, 117.0, 114.4, 44.3, 44.29, 38.0, 37.9, 30.8, 30.79, 28.7, 28.6, 24.1,24.0, 23.07, 23.05, 14.1, 10.63, 10.61.

#### 2.2.2. Synthesis of [Ph5FQPhO-diEH-PDI] Polymer

In a 5 mL flame-dried round-bottom flask filled with argon and equipped with a reflux condenser and a magnetic stirrer, vinyl-Ph5FQ-PhO-di-EH-PDI (100 mg, 0.0862 mmol) and AΙΒΝ (1.4 mg, 0.00862 mmol) were added and dissolved in 2 mL DMF_dry_. The system was degassed, flushed with argon again, and was heated at 120 °C for 3 days under argon. After the mixture was cooled, it was poured in a mixture of H_2_O/methanol, filtered, and washed with methanol, acetone, diethyl ether, and ethyl acetate. The obtained solid was dried under vacuum at 40 °C. The residual solid was dissolved in tetrahydrofuran in a 5 mL flame-dried round-bottom flask and refluxed for 4 h. The mixture was filtered and washed with tetrahydrofuran. The obtained solid was dried under vacuum at 40 °C overnight. Yield: 0.010 g (10%).

#### 2.2.3. Synthesis of [Ph5FQPhO-diEH-PDI]-[Ph5FQ] Polymer

In a 10 mL flame-dried round-bottom flask filled with argon and equipped with a reflux condenser and a magnetic stirrer, PPh5FQ (20 mg, 0.046 mmol), Ph5FQ-PhO-di-EH-PDI (100 mg, 0.0862 mmol), and K_2_CO_3_ (17.4 mg, 0.126 mmol) were added and dissolved in 3 mL DMF_dry_. The system was degassed, flushed with argon again, and was heated at 120 °C for 2 days under argon. After the mixture was cooled, it was poured in a mixture of H_2_O/methanol, filtered, and washed with acetone, diethyl ether, and ethyl acetate. The obtained solid was dissolved in tetrahydrofuran in a 20 mL flame-dried round-bottom flask and refluxed for 4 h. The mixture was filtered and washed with tetrahydrofuran. The obtained solid was dried under vacuum at 40 °C overnight. Yield: 0.050 g (50%).

### 2.3. Characterization Methods

^1^H, ^13^C, and ^19^F nuclear magnetic resonance (NMR) spectra were recorded on a Bruker Advance (Bruker BioSpin GmbH, Magnet Division, Karlsruhe, Germany) DPX 600.13, 150.90 MHz, and 564.69 MHz spectrometer, respectively, with CDCl_3_ or TCE-*d_4_* as solvents, containing TMS as internal standard.

Gel permeation chromatography (GPC) measurements were carried out using a Polymer Lab chromatographer (Agilent Technologies, Santa Clara, CA, USA) equipped with two pLgel 5 μm mixed columns and a UV detector, using CHCl_3_ as eluent with a flow rate of 1 mL/min at 25 °C calibrated versus polystyrene standards.

Attenuated total reflectance (ATR) spectra were recorded on a “Bruker Optics” Alpha-P Diamond ATR Spectrometer of Bruker Optics GmbH” (Ettlingen, Germany).

UV–Vis spectra were recorded using a Hitachi U-1800 spectrophotometer (Hitachi High-Technologies Europe GmbH, Mannheim, Germany). Continuous wave photoluminescence was measured using a Perkin Elmer LS50B spectrofluorometer (Waltham, MA, USA). All UV–Vis and PL measurements were performed in air using quartz cuvettes and flat quartz substrates for the examination of solutions and films, respectively. For the solution measurements, 10^−4^ M solutions in *o*-DCB were employed.

The electrochemical behavior of the fabricated materials was investigated using cyclic voltammetry (CV thereafter). CV experiments were carried out in a three-electrode cell. A glass substrate coated with fluorine-doped tin oxide (FTO) as a working electrode, a Pt wire secondary electrode, and a saturated Ag/AgCl reference electrode were used in the cell. Thin films of the fabricated materials were drop casted on FTO-coated glass slides, (Rsheet~8 Ω/square), and preheated at 80 °C for 20 min, from precursor solutions in chloroform. The resulting films were further annealed at 80 °C for 15 min. An Autolab PGSTAT 302 N electrochemical analyzer connected to a personal computer running the NOVA 1.8 software was used for data collection and analysis. All experiments were carried out at a scan rate of 0.1 V/s. Tetrabutylammonium hexafluorophosphate (TBAPF_6_) 0.1 M of anhydrous acetonitrile (CH_3_CN) was used as supporting electrolyte. Before carrying out the measurements, the cell was purged with pure argon for 20 min to remove diluted gasses. The reference electrode potential was calibrated against Ferrocene/Ferrocenium (Fc/Fc+) after each voltammetry run.

The LUMO energy levels were calculated from the first reduction onset potential using the following equation:

(1) $$E_{\mathrm{LUMO}}=e\;(E_{\mathrm{on}}^{\mathrm{RED}}-E_{1/2}^{\mathrm{Ferrocene}})-5.2\;[\mathrm{eV}]$$

$E_{\mathrm{on}}^{\mathrm{RED}}$ = the onset determined for the reduction peak of each molecule in cyclic voltammetry (V) versus Ag/AgCl.

$E{(}_{1/2}^{\mathrm{Ferrocene}}$) = ($E_{\mathrm{red}}+E_{\mathrm{OX}})/2\;$vs. Ag/AgCl.

The HOMO energy levels were obtained using the following equation:

(2) $$E_{g}^{\mathrm{opt}}=\mathrm{HOMO}-\mathrm{LUMO}$$

The $E_{g}^{\mathrm{opt}}$ (optical bandgap) was determined using the following equation:

(3) $$E_{g}^{\mathrm{opt}}=\frac{1240\;\left[\mathrm{eV}\;\mathrm{nm}\right]}{\lambda \mathrm{onset}\;\left[\mathrm{nm}\right]}\;[\mathrm{eV}]$$

Transmission electron microscopy (TEM) measurements were performed on a JEOL JEM2100 operating at 200 kV. Sample preparation for TEM examination involved the preparation of 10^−7^ M dilute solutions of the samples in *o-*DCB and filtration through a 0.45 μm filter. A drop of the solution was placed on 3 mm carbon coated copper grids (Electron Microscopy Sciences), and the samples were dried in air for 2 days. 

## 3. Results

### 3.1. Synthesis of PDI-Modified Perfluronophenyl Quinoline Polymers

A functional hydroxyphenyl-PDI derivative, named PhOH-di-EH-PDI, was recently reported by our group, prepared via the attachment of a hydroxyphenyl group to one bay position of the perylene diimide core [47]. The above molecule can be easily synthesized in multigram scale quantities via the Suzuki coupling reaction of mono-nitro PDI as an electrophile partner and the 4-(2-tetrahydropyranyloxy) phenylboronic acid, opening a new horizon for the preparation of large-scale PDI-based building blocks for organic electronics [52]. In that earlier report, the phenol functionality reacted with the “para” fluorine of 6-phenyl-(2-perfluorophenyl)-4-phenyl-quinoline (Ph5FQ), under basic conditions of nucleophilic substitution, affording ether-based PDI under mild conditions and in nearly quantitative yield. 

**Scheme 1:**
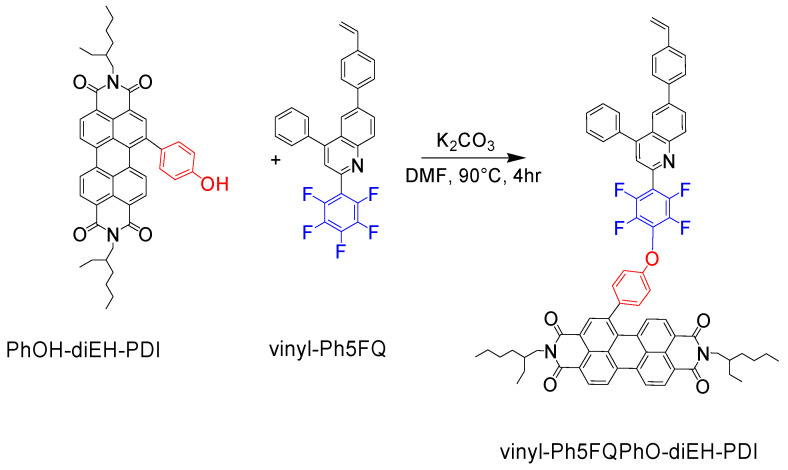
Synthesis of the vinyl-Ph5FQPhO-diEH-PDI monomer.

Herein, this methodology was extended even further using a polymerizable perfluorophenyl monomer or a preformed polymer backbone bearing perfluorophenyl side groups. In particular, we investigated the decoration of a vinyl functionalized quinoline derivative with PDI moieties, namely, the 6–vinylphenyl-(2-perfluorophenyl)–4–phenylquinoline (vinyl-Ph5FQ) [49]. The PhOH-di-EH-PDI reacted with vinyl-Ph5FQ using potassium carbonate (K_2_CO_3_) as a base in the dimethylformamide solvent (Scheme 1). We should note that the reaction’s temperature was no higher than 90 °C in order to keep the double bonds “alive”, and the reaction was completed in just 4 h. The final molecule, named vinyl-Ph5FQPhO-diEH-PDI, was isolated after purification with column chromatography in good yields of up to 85%. The vinyl-Ph5FQPhO-diEH-PDI presented excellent solubility in common organic solvents like toluene, tetrahydrofuran, and chloroform, and thus, it could be characterized via ^1^H NMR, ^19^F NMR, and ^13^C NMR spectroscopy.

**Figure 1:**
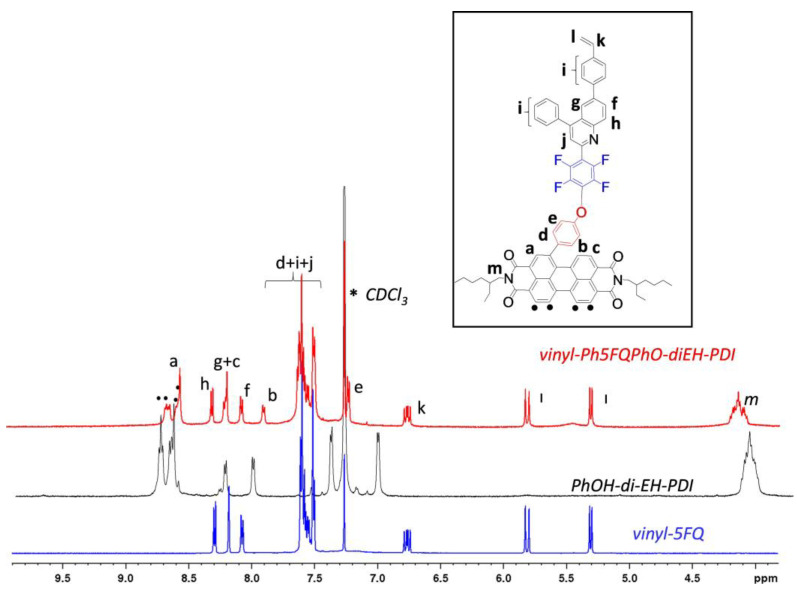
^1^H ΝΜR spectra (enlarged region 4.0–10.0 ppm) of vinyl-Ph5FQ (blue), PhOH-di-EH-PDI (black), and vinyl-Ph5FQPhO-diEH-PDI (red) in CDCl_3_. (The residual peaks of the solvent are denoted with (*)).

The ^1^H NMR spectra of vinyl-Ph5FQ, PhOH-di-EH-PDI, and vinyl-Ph5FQPhO-diEH-PDI are shown in Figure 1, (enlarged region 4.0–10.0 ppm). For the vinyl-Ph5FQPhO-diEH-PDI monomer, the signals are attributed to the corresponding protons marked with letters. By comparing the latter with the ^1^H NMR spectra of the two precursors (PhOH-di-EH-PDI and vinyl-Ph5FQ), the successful synthesis can be confirmed. Notably, the proton signals at 6.75, 5.80, and 5.30 ppm assigned to the vinyl group were not affected. The ^13^C NMR spectrum of the monomer vinyl-Ph5FQPhO-diEH-PDI is shown in Figure S1.

**Scheme 2:**
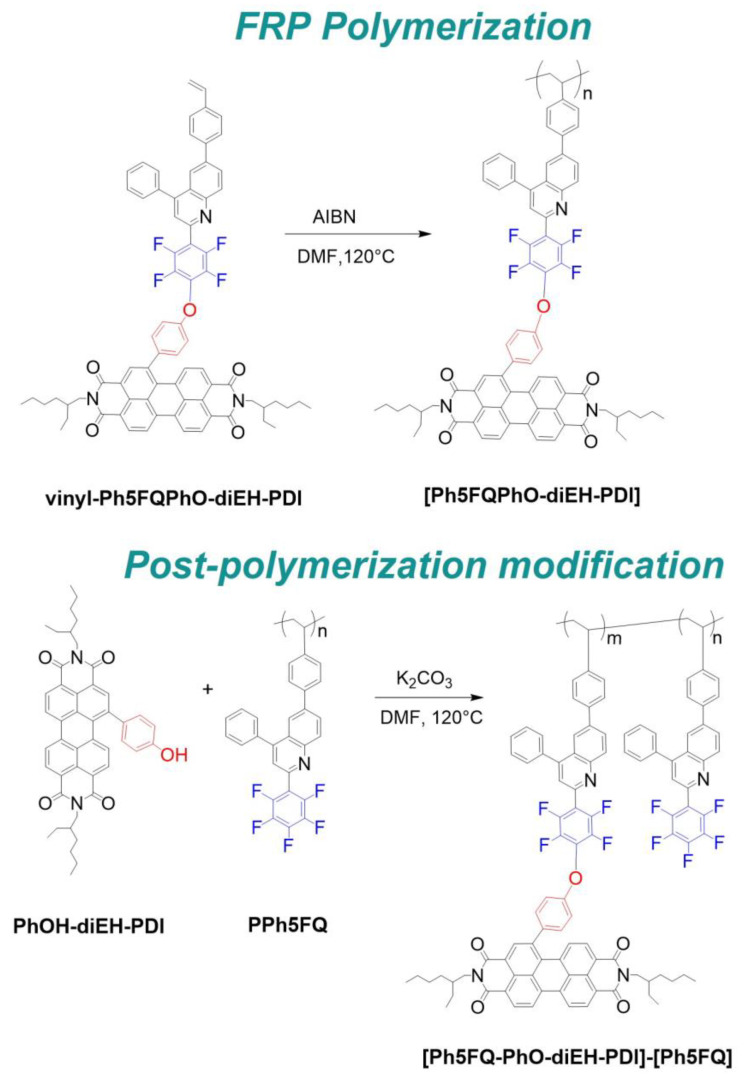
Synthesis of [Ph5FQPhO-diEH-PDI] and [Ph5FQPhO-diEH-PDI]-[Ph5FQ].

In the next step, the vinyl-functional PDI molecule was employed in free radical homopolymerization (Scheme 2), using AIBN as the initiator for the synthesis of the [Ph5FQPhO-diEH-PDI]. The crude product was extensively purified via treatment with various solvents such as ethyl acetate, diethyl ether, toluene, and tetrahydrofuran to ensure the successful removal of unreacted monomers or the presence of oligomers. However, the yield of the polymerization was found to be low, which is probably due to the strong stereochemical inhibition that the PDI skeleton induces as a side group, and the strong electron-withdrawing character of the PDI-bearing vinyl-5FQ.

In an alternative strategy, the nucleophilic substitution of the hydroxyphenyl-PDI derivative with the polymeric backbone of poly(vinyl-phenyl-(2-perfluorophenyl)–4–phenylquinoline) homopolymer (PPh5Q) was also studied for the development of perflurophenyl quinoline polymers decorated with PDI moieties. The synthesis of the homopolymer PPh5Q via FRP polymerization [49] afforded polymers of medium molecular weights of around ~8 kDa. Following this, the homopolymer reacted with the PhOH-di-EH-PDI moiety for the fluorine substitution at the para position. The modification was carried out under the same nucleophilic substitution conditions as the monomer vinyl-Ph5FQ, but this time, a higher temperature of 120 °C could be reached. The crude reaction product was treated with various solvents such as ethyl acetate, diethyl ether, and toluene to remove any residual monomers. The ^1^H NMR spectroscopy of the final PDI-bearing polymer revealed that not all fluorines in the para position were substituted. Thus, the final structure is defined as a copolymer named [Ph5FQPhO-diEH-PDI]-[Ph5FQ]. To our favor, this partial substitution with the PDI moiety and, thus, the absence of the extended PDI-containing blocks, led to better solubilities of the final copolymer, allowing for its structural elucidation via ^1^H NMR and ^19^F NMR analysis with CDCl_3_ as the solvent.

The evaluation of the polymers’ molecular weights was performed using gel-permeation chromatography (GPC) with chloroform at RT as the eluting solvent versus polystyrene standards. In Table 1, the molecular weights of the synthesized polymers are presented, while the GPC curves are shown in Figure S2. The Mn for the [Ph5FQPhO-diEH-PDI] homopolymer was estimated to be about 6 kDa. For the [Ph5FQ]-[Ph5FQPhO-diEH-PDI] copolymer, the Mn was estimated to be 16 kDa, while for the unmodified polymer PPh5FQ, it was found to be around 8 kDa, as mentioned above. The molecular weights obtained via GPC are possibly underestimated in the chloroform solvent, which is only a moderate solvent for the above PDI-decorated homopolymer, due to the differentiated structure of the polymers compared to the polystyrene standards. Especially for the [Ph5FQPhO-diEH-PDI] homopolymer that is slightly soluble in chloroform, we can assume that only the lower molecular weight polymer chains are detected. For the [Ph5FQ]-[Ph5FQPhO-diEH-PDI] copolymer, the para-fluoro substitution caused the GPC-measured size distributions to shift to higher molecular weights due to the increase in the size of the building block, without significant changes in the polydispersity index with respect to the original PPh5FQ polymer.

**Figure 2:**
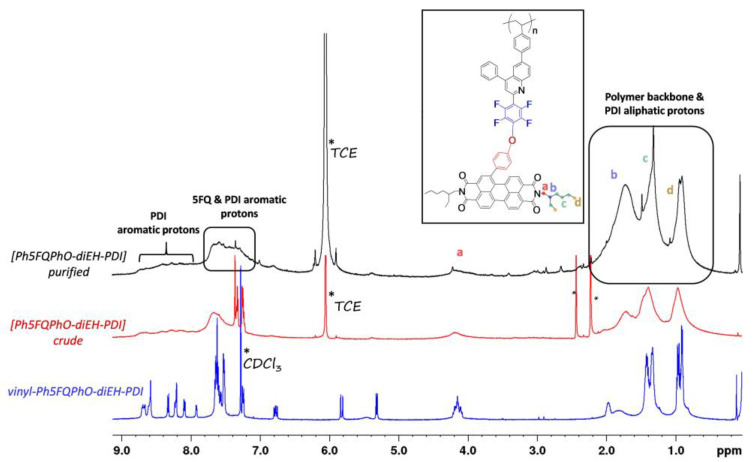
^1^H ΝΜR spectra of the vinyl-Ph5FQPhO-diEH-PDI (blue) in CDCl_3_, the crude homopolymer [Ph5FQPhO-diEH-PDI] before its purification using THF (red), and the final purified [Ph5FQPhO-diEH-PDI] (black), both in ΤCE-*d_4_*. (The residual peaks of the solvent are denoted with (*)).

In Figure 2, the ^1^H NMR spectra of the vinyl-Ph5FQPhO-diEH-PDI and of the [Ph5FQPhO-diEH-PDI] before and after purification are given. Due to the limited solubility of the polymer in common organic solvents, ^1^H NMR spectroscopy was performed in deuterated 1,1′,2,2′-tetrachloroethane (TCE-*d_4_*). Sharp peaks were initially observed between 7.2 and 7.5 ppm in the crude polymer spectrum, which are attributed to the aromatic protons of the monomer precursor vinyl-Ph5FQPhO-diEH-PDI or of the oligomers, e.g., dimers and trimers, produced. These were successfully removed after the extensive washing of the crude polymer with THF under reflux conditions. In the final purified [Ph5FQPhO-diEH-PDI] polymer, the signals of any vinyl bonds or PDI-oligomer peaks completely disappeared, and the characteristic aromatic protons assigned to the perylene skeleton appeared as a broad peak between 8.50 and 9.00 ppm. Also, the methylene protons of the ethylhexyl aliphatic chain of 4.0–4.3 ppm confirm the presence of the PDI in the polymeric structure.

**Figure 3:**
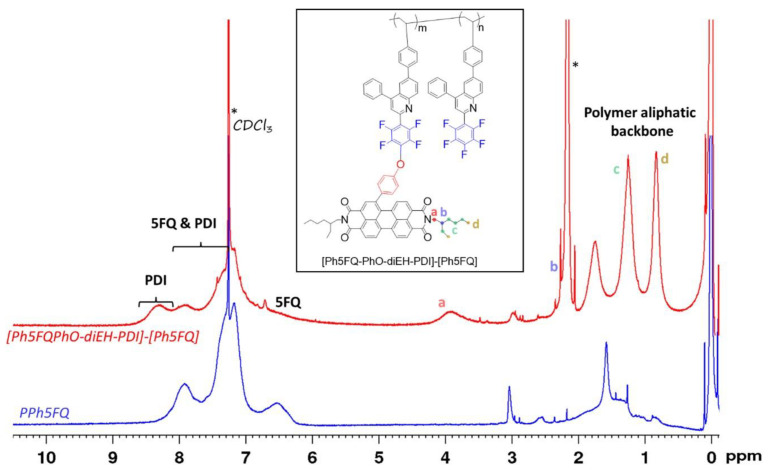
^1^ H ΝΜR spectra of the initial PPh5FQ homopolymer (blue) and of the PDI-modified polymer, namely, the [Ph5FQPhO-diEH-PDI]-[Ph5FQ] copolymer (red) in CDCl_3_. (The residual peaks of the solvent are denoted with (*)).

In Figure 3, the ^1^H NMR spectra of the original PPh5FQ polymer and of the PDI-modified [Ph5FQPhO-diEH-PDI]-[Ph5FQ] polymer are shown. The latter presents the characteristic PDI peaks at 4.0–4.3 ppm, assigned to the methylene aliphatic protons of the ethylhexyl imide group, and at 8.5–9.0 ppm, attributed to the PDI aromatic skeleton, confirming its successful attachment. From the integration of the peaks at the aromatic region and of the peak at 4.0–4.3 ppm assigned to the methylene aliphatic protons of the ethylhexyl imide group, a 40% content of the PDI-substituted Ph5FQ group was calculated in the final copolymer. Notably, as mentioned above, the copolymer [Ph5FQPhO-diEH-PDI]-[Ph5FQ] could be easily solubilized in CDCl_3_, in contrast to the [Ph5FQPhO-diEH-PDI] homopolymer, as shown in Figure 2.

**Figure 4:**
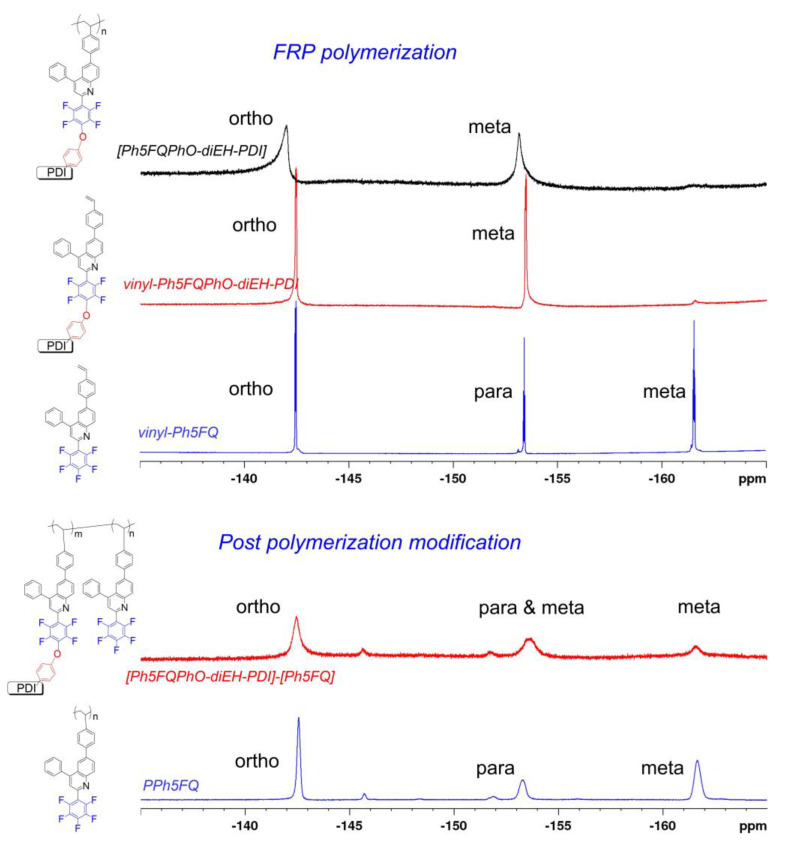
^19^F ΝΜR spectra of the vinylPh5FQ, vinylPh5FQPhO-diEH-PDI in CDCl_3_, and the [Ph5FQPhO-diEH-PDI] homopolymer in TCE-*d_4_*. For the post-modification procedure, the ^19^F ΝΜR spectra of PPh5FQ and [Ph5FQ]-[Ph5FQPhO-diEH-PDI] copolymer were both in CDCl_3_.

Moreover, ^19^F NMR spectroscopy was performed to investigate the substitution pattern of the perfluorophenyl ring. As shown in Figure 4, the ^19^F NMR spectra of the non-substituted vinyl-Ph5FQ presented three sharp peaks at −142.45, −153.38, and −161.57 ppm assigned to the fluorine atoms at the ortho, para, and meta positions of the perfluorophenyl group, respectively. In the ^19^F NMR spectra of the PDI-functionalized monomer, two double of doublet signals of equal proportion are observed at −142.45 ppm and −153.48 ppm, concluding that the reaction proceeded regioselectively at the para position. The specific chemical shift of fluorines for the perfluorophenyl group are also supported by similar cases in the literature in which the perflurophenyl group acts as a functionality agent for nucleophilic substitutions [41,53]. For the homopolymer [Ph5FQPhO-diEH-PDI], the two different types of fluorine atoms (ortho and meta) are also present, which is in line with the ^19^F NMR analysis of the vinyl-Ph5FQPhO-diEH-PDI. For the [Ph5FQPhO-diEH-PDI]-[Ph5FQ] copolymer, the partial substitution of fluorines in the para position is also evident in the ^19^F NMR spectra. From the integration of the ^19^F NMR spectra peaks, a 40% content of PDI-substituted Ph5FQ groups is calculated in the final copolymer, which is in agreement with the ^1^H NMR spectra calculation. Notably, further modification percentages were not achieved, even after prolonged post-polymerization modification reaction times. Therefore, it is concluded that the modification degree was limited to 40%, only due to stereochemical reasons.

Finally, all of the polymers were analyzed via ATR spectroscopy (Figure S3). Characteristic absorption bands of PDI (1695 and 1655 cm^−1^ (C=O imide)), (2955 and 2924 cm^−1^ C-H stretching of aliphatic chains), and (1590 and 1505 cm^−1^ aromatic) are observed at the ATR spectra of the PDI-modified polymers. Also, new bands appeared at 1320 and 1240 cm^−1^, which were assigned to the aromatic stretching of the ether linkages, providing additional evidence of the successful aromatic ether bond formation.

### 3.2. Optoelectronic Characterization

**Figure 5:**
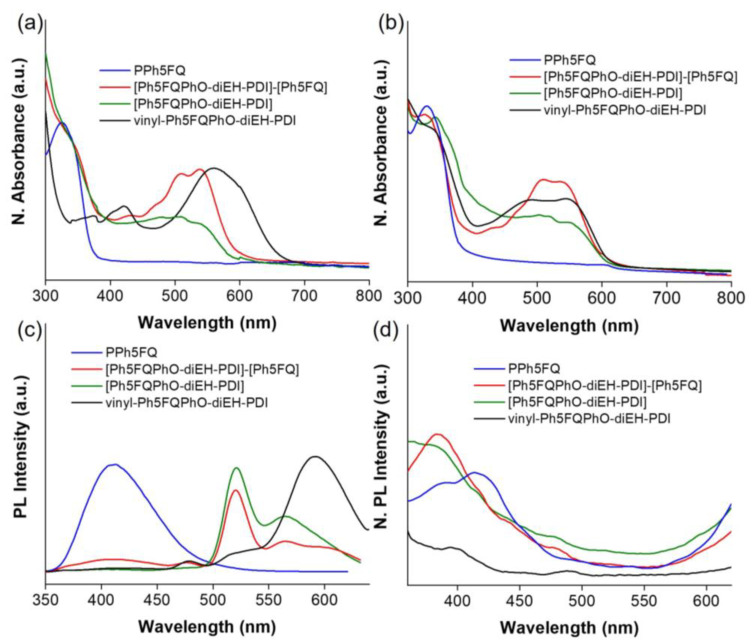
Normalized absorbance of PPh5FQ, vinyl-Ph5FQPhO-diEH-PDI, [Ph5FQPhO-diEH-PDI]-[Ph5FQ], and [Ph5FQPhO-diEH-PDI] (**a**) in *ο*-DCB solutions and (**b**) in film form. Normalized PL intensity of PPh5FQ, vinyl-Ph5FQPhO-diEH-PDI, [Ph5FQPhO-diEH-PDI]-[Ph5FQ], and [Ph5FQPhO-diEH-PDI] (**c**) in *ο*-DCB solutions and (**d**) in film form.

The optical properties of the synthesized vinyl-Ph5FQPhO-diEH-PDI monomer and of the PPh5FQ, [Ph5FQPhO-diEH-PDI], and [Ph5FQPhO-diEH-PDI]-[Ph5FQ] polymers were evaluated using UV–Vis and PL spectroscopies (Figure 5). All synthesized materials were studied in *ortho*-dichlorobenzene (*o*-DCB) solutions and in a solid state by preparing thin films via the drop casting of dilute solutions. The optical band gaps were determined based on the absorption onsets using Equation (3).

The UV–Vis spectra of the above materials in the *o*-DCB solutions are presented in Figure 5a. In all cases, the characteristic absorption peak of the perfluorophenyl quinoline with a maximum absorbance at 330 nm appears. After the substitution of the para fluorine atom with the PDI unit, an intense absorption peak in the range of 450–650 nm is evident. For the vinyl-Ph5FQPhO-diEH-PDI monomer, an intense broad absorbance peak between 500 and 650 nm coexists, which is attributed to the strong electronic interactions of the electron-deficient perylene core with the electron-donating character of the ether group [54,55]. For the [Ph5FQPhO-diEH-PDI] polymer, the absorption peak associated with the perflurophenyl quinoline group at 330 nm is maintained, but the absorbance peak attributed to the PDI group appeared to diminish, which is attributed to the strong aggregation tendency of the PDI chromophores. It is possible that intense intra and inter p-p stacking occurs among the PDI moieties within the polymer chain itself or between the PDI units of different chains because of intramolecular aggregation. Since the PDI moieties are covalently bound in proximity along the polymer backbone, this leads to high local concentrations and, in turn, to intense aggregation phenomena [56].

For the copolymer [Ph5FQPhO-diEH-PDI]-[Ph5FQ] with both PDI-substituted and non-substituted perflurophenyl quinoline groups, the absorbance at the visible region related with the absorbance of PDI is not drastically affected. This fact is explained by weaker interactions among the PDI units that are distributed in greater distances along the polymer backbone, resulting in less stereochemical inhibition in the final polymeric structure. Similar absorption behavior appeared for the films, with redshifted absorption peaks related to the formation of aggregates or stronger solid-state interactions for the [Ph5FQPhO-diEH-PDI] homopolymer in comparison to the [Ph5FQPhO-diEH-PDI]-[Ph5FQ] copolymer (Figure 5b). The absorption of the substituted polymers with the PDI moieties is an approximate superposition of that of the perflurophenyl quinoline and PDI, suggesting that there is no ground state charge transfer in the polymers system.

The photoluminescence spectra (PL) of the vinyl-Ph5FQPhO-diEH-PDI and of the PPh5FQ, [Ph5FQPhO-diEH-PDI], and [Ph5FQPhO-diEH-PDI]-[Ph5FQ] in the *o*-DCB solutions and in the solid state are shown in Figure 5c and 5d, respectively. The PPh5FQ presents an emission maximum at 430 nm, which is typical for quinoline polymers. With the introduction of PDI, and upon excitation of the vinyl-Ph5FQPhO-diEH-PDI and its homopolymer [Ph5FQPhO-diEH-PDI] at 330 nm, both present complete quenching of this emission peak at 410 nm, and a new emission appears. The [Ph5FQPhO-diEH-PDI] presents a dual emission band with maxima at 520 and 570 nm, and the vinyl-Ph5FQPhO-diEH-PDI shows a more redshifted emission with a maximum at 600 nm, which is in agreement with their absorption profiles. For the [Ph5FQPhO-diEH-PDI]-[Ph5FQ], a similar emission profile with the [Ph5FQPhO-diEH-PDI] is observed with an additional small emission peak at 410 nm due to the unsubstituted perflurophenyl groups.

**Figure 6:**
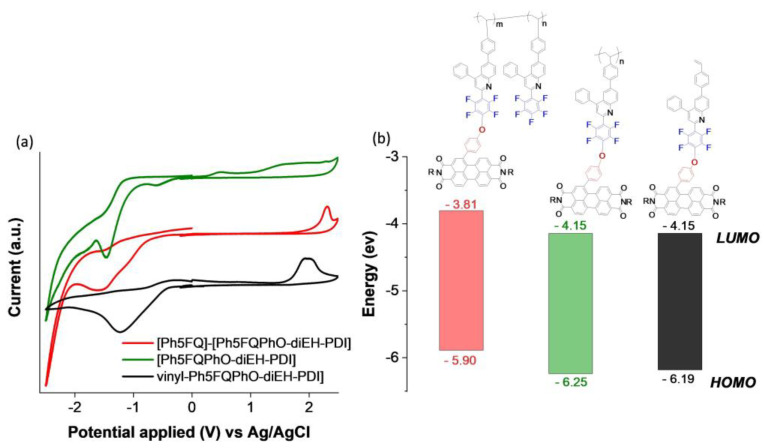
(**a**) Cyclic voltammograms of vinyl-Ph5FQPhO-diEH-PDI, [Ph5FQPhO-diEH-PDI]-[Ph5FQ], and [Ph5FQPhO-diEH-PDI] from −2.6 V to 2.6 V and (**b**) calculated energy levels.

The electrochemical properties of all synthesized materials were investigated via cyclic voltammetry (CV). The experiments were performed in film form, in CH_3_CN in the presence of Bu_4_NPF_6_, as supporting electrolyte. The lowest unoccupied molecular orbital (LUMO) energy levels were determined by the reduction onsets. The highest occupied molecular orbital (HOMO) energy levels were estimated using Equations (2) and (3). The calculated $E_{g}^{\mathrm{opt}}$, LUMO, and HOMO levels are given in Table 2, and the cyclic voltammograms of the materials are shown in Figure 6.

In Figure 6, two reduction peaks are observed for the [Ph5FQPhO-diEH-PDI]. The first, with an onset at −0.50 V, attributed to the PDI’s reduction, and the second, with an onset at −1.00 V, attributed to perfluorophenyl quinoline, are in good agreement with the reduction profiles of these molecules according to previous studies [46]. For the vinyl-Ph5FQPhO-diEH-PDI monomer, the first reduction onset is also determined to be −0.50 V, and the LUMO level is calculated to be −4.15 V. The [Ph5FQPhO-diEH-PDI]-[Ph5FQ] copolymer bearing unsubstituted quinoline groups presents the combined reduction peaks of quinoline and PDI, with the fist reduction onset at −0.85 V and the LUMO level of −3.81 eV. This result is reasonable, as the free perfluorophenyl quinoline group is expected to increase the LUMO level due to its weaker electron-accepting character compared to the PDI [48].

### 3.3. Morphological Characterization

**Figure 7:**
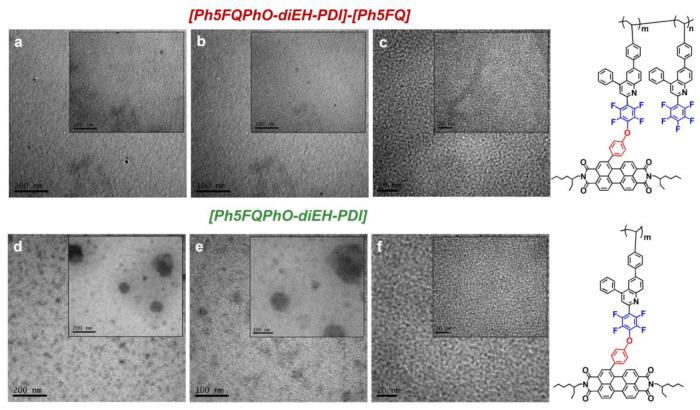
TEM images of the [Ph5FQPhO-diEH-PDI]-[Ph5FQ] copolymer at (**a**) 200 nm, (**b**) 100 nm, and (**c**) 20 nm (**top** images), and of the [Ph5FQPhO-diEH-PDI] homopolymer at (**d**) 200 nm, (**e**) 100 nm, and (**f**) 20 nm (**bottom** images). The images in insets correspond to the annealed films at 120 °C for the respective magnification.

The thin film morphology of the two PDI-substituted perflurophenyl quinoline polymers was probed via transmission electron spectroscopy (TEM). The TEM images (Figure 7) were obtained by drop casting the corresponding specimen on the TEM sample grid from very dilute *o*-DCB solutions of approximately 10^−7^ M concentrations. The [Ph5FQPhO-diEH-PDI]-[Ph5FQ] presented homogeneous thin films with extended nanophase separation but without extended aggregate formation. Only some darker areas of circular formations of about 15 nm in size appeared, but in no case were there extended aggregates. Upon thermal annealing at 120 °C for 10 min of the [Ph5FQPhO-diEH-PDI]-[Ph5FQ] copolymer film, the uniformity was preserved. The only notable feature was that circular structures like those prior to the heat treatment were also present. In the bottom row, the TEM images for the homopolymer [Ph5FQPhO-diEH-PDI] are provided in the same magnifications and under the same thermal treatment conditions. The homopolymer displays large darker formations, which is possibly owing to the formation of aggregates of the PDI moieties attached along every single polymeric unit. These formations appeared to be even larger and more intense after thermal annealing that obviously led to the enhanced aggregation of the PDI moieties.

## 4. Conclusions

The modification of perflurophenyl quinoline-based monomer and polymer was studied through a simple reaction of nucleophilic aromatic substitution of the para fluorines of the perflurophenyl group via a phenol-functionalized PDI derivative. This methodology was successfully tested for both the vinyl-perflurophenyl-quinoline monomer and the perflurophenyl quinoline homopolymer as a post-polymerization modification route. The substitution of the PDI unit was evaluated through ^1^H, ^13^C NMR, and ^19^F NMR spectroscopies, providing distinguished and well-separated resonances. Through the post-polymerization modification route, perflurophenyl quinoline polymers partially substituted with PDI were provided, whereas the homopolymerization of PDI-bearing vinyl-perflurophenyl quinoline molecule afforded a fully PDI-substituted perflurophenyl quinoline polymer. The final polymers, due to this structural difference, presented different optical and electrochemical characteristics as well as different morphological features that were investigated using TEM microscopy. The modification through the “active” PDI molecules as nucleophilic agents can also be readily applied in other polymeric systems, altering their properties toward more complex, fine-tuned materials. Thus, this nucleophilic substitution approach shows a new pathway toward functional semiconducting materials and the fabrication of diverse sets of tailor-made polymer semiconductors.

## Figures and Tables

**Table 1:** GPC analysis ^1^ of the initial PPh5FQ, [Ph5FQ]-[Ph5FQPhO-diEH-PDI], and [Ph5FQPhO-diEH-PDI] polymers.

Polymer	Mn ^2^	Mw ^2^	Đ ^2^
[Ph5FQPhO-diEH-PDI]	6200	7200	1.17
PPh5FQ	7800	11,400	1.46
[Ph5FQPhO-diEH-PDI]-[Ph5FQ]	15,900	23,000	1.45

^1^ Chromatograms were obtained at 25 °C, using chloroform as eluent versus PS standards. The UV detector was set at 254 nm. ^2^ Mn = number average molecular weight, Mw = mass average molecular weight, and Đ = dispersity.

**Table 2:** Electrochemical properties of the synthesized materials.

Polymer	λmax (nm)			$E_{g}^{opt}$ (eV)	$E_{on}^{RED}$ (V)	E_LUMO_ (eV)	E_HOMO_ (eV)
	Sol	Film	Onset				
vinyl-Ph5FQPhO-diEH-PDI	560	330/545	600	2.07	−0.50	−4.15	−6.19
[Ph5FQPhO-diEH-PDI]	325/510	345/510	590	2.10	−0.50	−4.15	−6.25
[Ph5FQ]-[Ph5FQPhO-diEH-PDI]	325/540	325/510	595	2.09	−0.85	−3.81	−5.90

## Data Availability

Experimental data are available from the authors upon reasonable request.

## Supplementary Materials

The following supporting information can be downloaded at https://www.mdpi.com/article/10.3390/polym15122721/s1, Figure S1: ^13^C ΝΜR spectra of vinyl-Ph5FQPhO-diEH-PDI; Figure S2: GPC traces of the precursor homopolymer PPh5FQ (black line), the [Ph5FQPhO-diEH-PDI]-[Ph5FQ] copolymer (red line) and of the [Ph5FQPhO-diEH-PDI] homopolymer (green line), using CHCl_3_ as eluent at a flow rate of 1 mL/min with the UV detector set at 254 nm, at 25 °C; Figure S3: ATR spectra of the precursor homopolymer PPh5FQ, of the [Ph5FQPhO-diEH-PDI]-[Ph5FQ] copolymer and of the [Ph5FQPhO-diEH-PDI] homopolymer.
