# Supplementary material for: Nucleophilic Aromatic Substitution of Pentafluorophenyl-Substituted Quinoline with a Functional Perylene: A Route to the Modification of Semiconducting Polymers
Source: Polymers (Basel). 2023 Jun 18;15(12):2721. doi: 10.3390/polym15122721 (PMC10301806; doi:10.3390/polym15122721)
Supplement: Supplementary file 1 [file polymers-15-02721-s001.zip › polymers-2459060-supplementary.pdf]

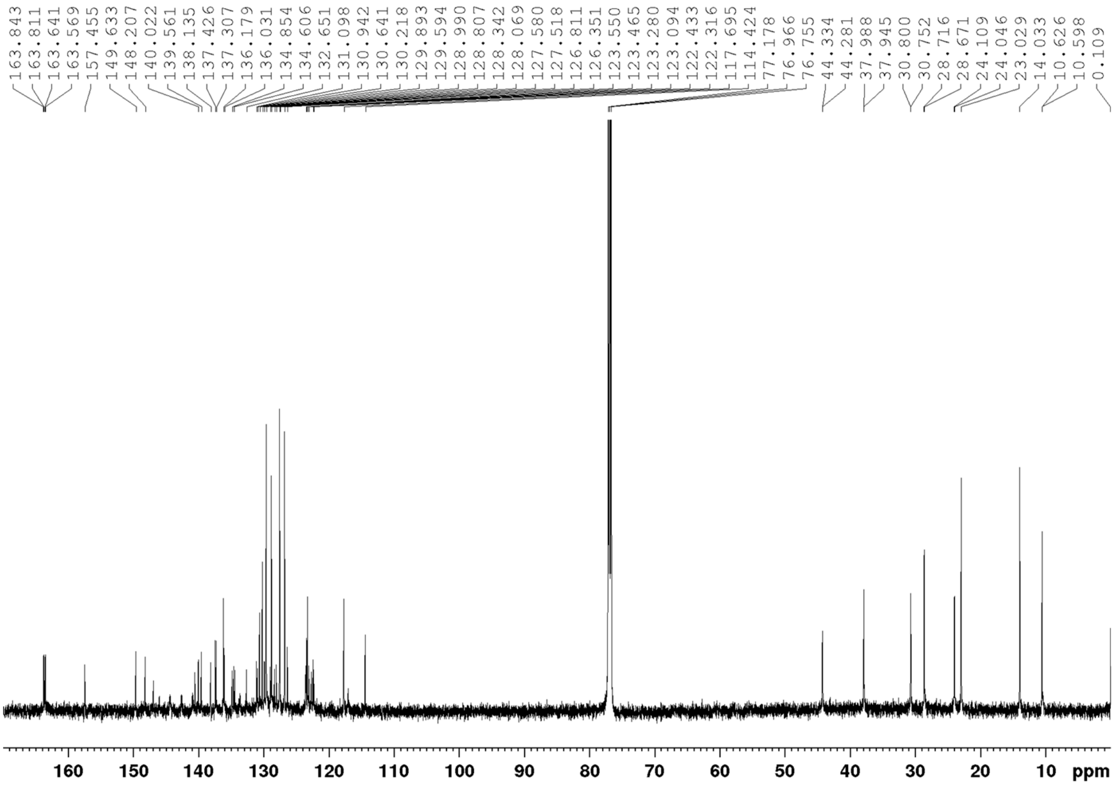

Figure S1:^{13}C NMR spectra of vinyl-Ph5FQPhO-diEH-PDI.

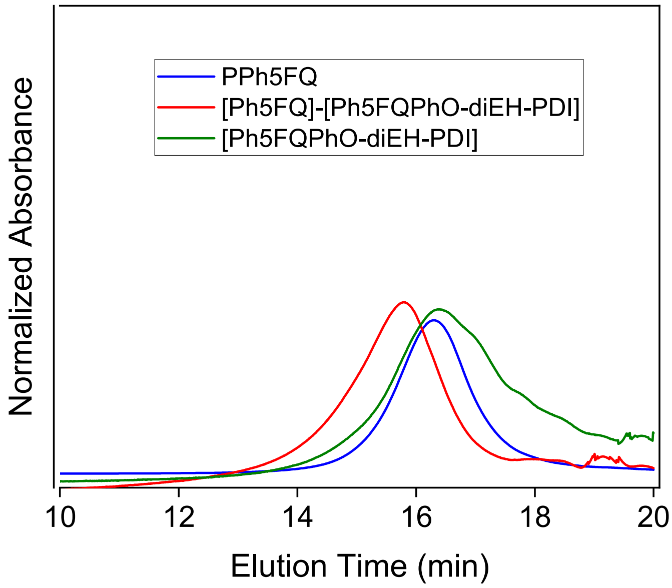

Figure S2. GPC traces of the precursor homopolymer PPh5FQ (black line), the [Ph5FQPhO-diEH-PDI]-[Ph5FQ] copolymer (red line) and of the [Ph5FQPhO-diEH-PDI] homopolymer (green line), using $CHCl_3$ as eluent at a flow rate of 1 mL/min with the UV detector set at 254nm, at 25°C.

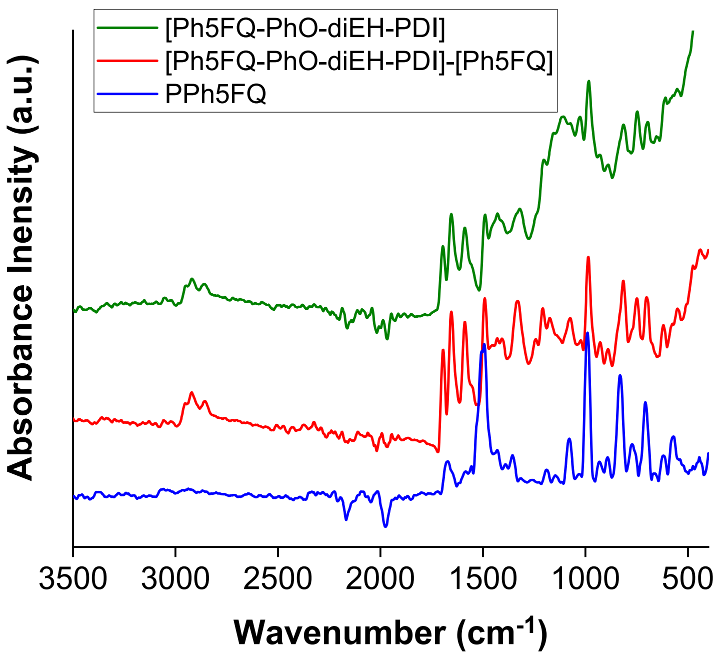

Figure S3. ATR spectra of the precursor homopolymer PPh5FQ, of the [Ph5FQPhO-diEH-PDI]-[Ph5FQ] copolymer and of the [Ph5FQPhO-diEH-PDI] homopolymer**.**
